# Supplementary material for: Validity and Applicability of the Global Leadership Initiative on Malnutrition (GLIM) Criteria in Patients Hospitalized for Acute Medical Conditions
Source: Nutrients. 2023 Sep 16;15(18):4012. doi: 10.3390/nu15184012 (PMC10535463; doi:10.3390/nu15184012)
Supplement: Supplementary file 1 [file nutrients-15-04012-s001.zip › nutrients-2563731-supplementary.pdf]

Common block	SGA block	GLIM block
- Weight 6 months ago as reported by the patient (in kg)<br>- Current weight (in kg)<br>- Calculation of % weight loss in the last 6 months, using the following formula: [Usual weight (kg) - current weight (kg)] / Usual weight (kg) x 100<br>- Digestive symptoms presented in the last few weeks, reported by the patient: nausea, vomiting, constipation, diarrhea, anorexia/hyporexia, dysphagia, or abdominal pain	- Changes in intake: inquired about changes in the diet, and if they occurred, assessed the duration and type of change.<br>- Modification in functional capacity: inquired about the loss of functional capacity, and if positive, about the degree and duration of it<br>- Increased metabolic demand based on the disease, for example, in the presence of active infection, trauma, or surgery.<br>- Manual examination to subjectively determine the presence or absence of an alteration, and if applicable, determine its degree (mild, moderate, or severe).:<br>- Subcutaneous fat loss: evaluated in the triceps area and the mid-axillary line<br>- Subcutaneous mass loss: evaluated in the quadriceps and deltoid areas<br>- Edema: evaluated in ankles and sacral area<br>- Ascites	- Current height (in cm)<br>- Calculation of BMI: Current weight (kg) / Height (m)^2<br>- Measurement of CC on the dominant leg: It was determined on the dominant side with the patient sitting (in the case of a patient unable to sit, it was performed in supine position) with the foot supported, the leg flexed at a 90º angle, and at the maximum CC. CC values were adjusted based on the BMI using the method described by Gonzalez *et al [19]*.<br>- Determination of the presence of conditions that may reduce nutrient absorption, such as irritable bowel syndrome, pancreatic insufficiency, gastric bypass, intestinal pseudo-obstruction, gastroparesis, chronic diarrhea, or steatorrhea.<br>- Current dietary intake in relation to usual intake. It was qualitatively assessed by questioning the patient regarding whether their intake in the last few weeks had been 0%, 25%, 50%, 75%, or 100% compared to their usual intake<br>- Determination of C-Reactive Protein value included in the patient's admission blood test.

Abbreviations: BMI = body mass index; CC = calf circumference
